# A pilot randomized controlled trial of the feasibility of a self-directed coping skills intervention for couples facing prostate cancer: Rationale and design

**DOI:** 10.1186/1477-7525-10-119

**Published:** 2012-09-26

**Authors:** Sylvie D Lambert, Afaf Girgis, Jane Turner, Patrick McElduff, Karen Kayser, Paula Vallentine

**Affiliations:** 1 Translational Cancer Research Unit, Ingham Institute for Applied Medical Research, South Western Sydney Clinical School, UNSW Medicine, The University of New South Wales, Liverpool, BC NSW, 2170, Australia; 2 The University of Queensland, Mental Health Centre K Floor, Herston, QLD, 4029, Australia; 3 School of Medicine and Public Health, The University of Newcastle, Hunter Medical Research Institute (HMRI) Building, New Lambton, NSW, 2305, Australia; 4 Kent School of Social Work, University of Louisville, Louisville, KY, 40292, USA; 5 Cancer Council NSW, 153 Dowling Street, Woolloomooloo, NSW, 2011, Australia

**Keywords:** Psychosocial adjustment, Couple, Family, Prostate cancer, Stress-coping, Dyadic coping, Anxiety, Intervention, Self-directed, Information resources

## Abstract

**Background:**

Although it is known both patients’ and partners’ reactions to a prostate cancer diagnosis include fear, uncertainty, anxiety and depression with patients’ partners’ reactions mutually determining how they cope with and adjust to the illness, few psychosocial interventions target couples. Those that are available tend to be led by highly trained professionals, limiting their accessibility and long-term sustainability. In addition, it is recognised that patients who might benefit from conventional face-to-face psychosocial interventions do not access these, either by preference or because of geographical or mobility barriers. Self-directed interventions can overcome some of these limitations and have been shown to contribute to patient well-being. This study will examine the feasibility of a self-directed, coping skills intervention for couples affected by cancer, called *Coping-Together,* and begin to explore its potential impact on couples’ illness adjustment. The pilot version of *Coping-Together* includes a series of four booklets, a DVD, and a relaxation audio CD.

**Methods/design:**

In this double-blind, two-group, parallel, randomized controlled trial, 70 couples will be recruited within 4 months of a prostate cancer diagnosis through urology private practices and randomized to: 1) *Coping-Together* or 2) a minimal ethical care condition. Minimal ethical care condition couples will be mailed information booklets available at the Cancer Council New South Wales and a brochure for the Cancer Council Helpline. The primary outcome (anxiety) and additional secondary outcomes (distress, depression, dyadic adjustment, quality of life, illness or caregiving appraisal, self-efficacy, and dyadic and individual coping) will be assessed at baseline (before receiving study material) and 2 months post-baseline. Intention-to-treat and per protocol analysis will be conducted.

**Discussion:**

As partners’ distress rates exceed not only population norms, but also those reported by patients themselves, it is imperative that coping skills interventions target the couple as a unit and enhance both partners’ ability to overcome cancer challenges. This pilot study will examine the feasibility and potential efficacy of *Coping-Together* in optimising couples’ illness adjustment. This is one of the first feasibility studies to test this innovative coping intervention, which in turn will contribute to the larger literature advocating for psychosocial care of couples affected by prostate cancer.

**Trial registration:**

Australian New Zealand Clinical Trials Registry ACTRN12611000438954

## 

In Australia, cancer is the leading cause of disease-related burden (accounting for nearly one-fifth of the total disease burden) [1]. In 2007, 108,368 new cases of cancer were diagnosed in Australia (excluding basal and squamous cell carcinomas of the skin), and slightly more than half of these cases were men [2]. Prostate cancer is the most common type of cancer among men with 19,403 cases diagnosed in 2007 [2] and 1 in 4 men now diagnosed by 85 years of age [3]. Nearly all patients who present with localised prostate cancer will live beyond five years with the 10- and 15-year survival rates being 93% and 77%, respectively [4]. As the number of people living beyond initial diagnosis is increasing, so is the time during which the disease sequelae and psychosocial consequences must be managed. Consequently, the focus in practice and research has shifted from viewing cancer as a terminal illness to helping patients and partners learn to cope with cancer demands and optimise functioning and quality of life.

Although treatment for prostate cancer is becoming increasingly successful, the initial diagnosis still comes as a shock [5,6] and resulting treatment often adversely impacts both patients’ and partners’ quality of life, including their social, psychological, and physical functioning [5-14]. Approximately one third of men diagnosed with prostate cancer report psychological distress [15]. In some contexts, wives of men with prostate cancer have reported as much, if not more, distress than patients [5,9,16]. Although Eton et al. [9] found that men with prostate cancer and their spouses did not differ in terms of general distress; the spouses reported more cancer specific distress than the patients. The concern is that a recent meta-analysis by Hagedoorn et al.[17] found a moderate, positive association between patients’ and partners’ levels of distress (r = .29, p < .001), which implies that patient’s high distress has an impact on their partner’s illness adjustment (or vice-versa). It follows from this that psychosocial interventions should then target *couples*[18-20].

The predictors of patient and partner anxiety are typically categorised into three broad classes of variables: demographics, characteristics/stages of disease and treatment, and psychosocial [12,13,21,22]. Amongst psychosocial variables, coping is arguably one of the most frequently studied predictors of patient and partner anxiety [12,13,23]. Although findings remain equivocal, most studies support that coping strategies directed toward active engagement with the stressor, including both problem- and emotion-focused coping, are associated with more positive adjustment [24]. In contrast, those considered less functional, such as avoidance, behavioural disengagement or denial, are associated with higher levels of distress [12,13,22,25,26]. Beyond individual approaches to coping, recent studies have examined the impact of each partners’ way of coping on the other’s stress (i.e., dyadic coping) and suggest that illness adjustment is enhanced when patients and partners are mutually responsive to each other’s stress, view the cancer challenges as “our” problem or “being in it together”, and are engaged in collaborative dyadic coping by pooling their resources and problem-solving jointly [27,28]. This evidence suggests that a couple-based intervention focused on maximising use of individual and dyadic adaptive coping skills might be most promising in reducing the psychological distress of patients and partners in response to a stressful situation like cancer [21].

Recently, reviews of couple-based interventions suggest that these have the potential to be as, if not more, efficacious than patient-only interventions in optimising patient adjustment and are more efficacious than usual care in enhancing partners’ adjustment [29]. For instance, Scott et al. [30] compared the efficacy of CanCOPE (a couple-based, coping skills training intervention for women diagnosed with breast or gynaecological cancer and their partners) with an individual coping intervention and a medical information only condition. CanCOPE women reported significantly less distress and less avoidance post-intervention than the women in the other two conditions. There was also a trend for CanCOPE partners to report less distress than those in the other conditions. Similarly, Nezu et al. [31] examined the efficacy of a problem-solving therapy (PST) among a mixed sample of patients diagnosed with cancer and included two treatment groups: one in which patients attended the PST alone and another one in which PST was attended with a significant other. Post-PST positive effects on quality of life and distress were similar in the two treatment groups. However, at 6- and 12-month follow-ups, patients participating in PST with their partner reported lower distress than patients who attended the PST alone. In the context of other chronic illnesses, couple-based coping or educational interventions have also been shown to be more efficacious than individual-level interventions in optimising illness adjustment [32].

Whilst couple-based interventions are promising in enhancing couples’ illness adjustment, most are delivered by highly trained health professionals [33], limiting their long-term accessibility and sustainability, due to high cost and limited availability of qualified professionals, especially in non-metropolitan areas. In addition, it is recognised that patients who might benefit from conventional face-to-face psychosocial interventions do not access these, either by preference or because of geographical or mobility barriers [34,35]. Studies have found that as few as 14% of distressed patients with cancer accept referral to psychosocial services [36]. Thus, the challenge is to develop a cost-effective couple-based intervention that is accessible, sustainable, and efficacious. One suggestion for developing a cost-effective approach to psychosocial interventions involves using a group format instead of an individual format [37]. However, research supporting the feasibility and efficacy of group interventions over individual ones remains equivocal [37,38]. Another suggestion for cost containment is the use of a self-directed (also referred to as self-help or self-administered) format [39].

Self-directed interventions overcome some of the barriers of face-to-face services and have several potential advantages for couples, including the choice of selecting what, when, and how they want to learn. In the general self-help literature, unguided, self-help interventions, either print- or computer-based, have been shown to enhance individual well-being, including decreasing anxiety and depression symptoms [40,41]. The few self-directed, coping skills interventions developed for individuals facing cancer have been found to be acceptable [42,43], efficacious in enhancing patient well-being [39,42], particularly among distressed individuals [44], and cost-effective [39]. In a feasibility study by Alison et al. [43], when patients were questioned about their preferred format for a coping skills intervention, 5% selected the group format, 56% selected the one-on-one format, and 39% selected the self-directed format. Interestingly, more men chose the self-directed format. Beatty et al. [42] recently found support for the efficacy of a self-directed, coping skills workbook for women with breast cancer in improving post-traumatic stress disorder symptoms, cognitive avoidance, and helplessness/hopelessness. The workbook contained information on common medical and psychosocial issues, cognitive behaviour therapy-based worksheets to enable active engagement and processing of these issues, and survivors’ stories. Interestingly, Krischer, Xu & Jacobsen [44] examined the efficacy of a self-administered stress management training intervention among individuals undergoing radiotherapy, including men diagnosed with prostate cancer, and found that distressed individuals benefited more from the self-administered intervention (improvements in mental health and decreased depressive symptoms) than non-distressed individuals. In another context, Dalal et al. [45] found that a self-directed manual was as effective as hospital-based rehabilitation for patients after myocardial infarction in increasing quality of life and reducing total cholesterol levels. The main limitation of the aforementioned self-directed interventions is the exclusive focus on patients, neglecting partners.

Hence, our team has recently developed a self-directed coping skills training intervention for couples affected by cancer, called *Coping-Together,* and this pilot study will examine the feasibility of *Coping-Together* and begin to explore its potential impact on couples’ illness adjustment. For the purpose of the pilot study, the following *Coping-Together* materials will be included: four booklets, a DVD featuring ‘communicating effectively with health care professionals’ and a relaxation audio CD.

## The *Coping-Together* intervention

### Theoretical and empirical underpinnings

*Coping-Together* is a novel, evidence-based, self-directed psychosocial intervention to help couples develop adaptive, individual and dyadic, coping skills and feel more confident in applying these to their current situation. *Coping-Together* builds on three main theoretical frameworks: 1) Lazarus & Folkman’s Stress and Coping framework [46], 2) Bodenmann’s framework of dyadic coping [47], and 3) Bandura’s self-efficacy theory [48].

**Figure 1:**
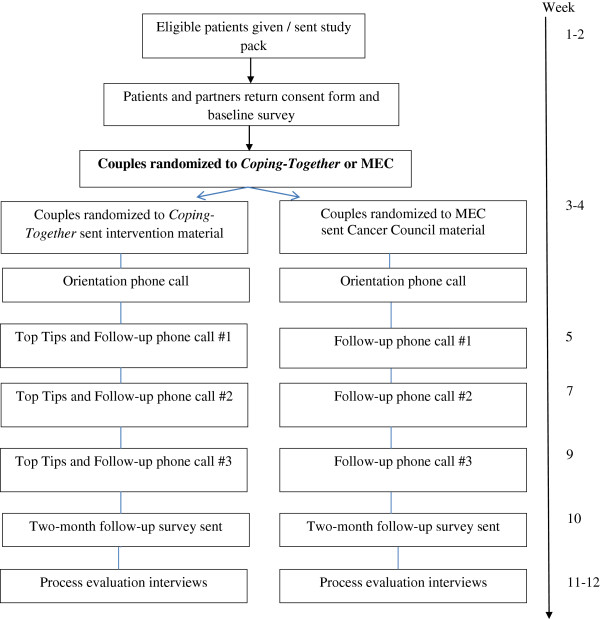
**How the *Coping-Together *intervention can enhance the coping process.**

Of the various coping with stress frameworks, the Lazarus & Folkman framework is the best known and most widely used in the study of stress caused by cancer. This framework explains that the coping process is initiated in response to the individual’s appraisal that important goals have been *harmed, lost, or threatened*[23]. Coping is typically characterised either as problem-focused coping (alter the stressful situation using strategies such as information-seeking, planning and problem-solving) or emotion-focused coping (regulate situation-related emotions using strategies such as positive reappraisal and behavioural disengagement) [23]. These coping styles are often further considered for their adaptive (e.g., positive reappraisal) versus maladaptive (e.g., denial) nature. The assumption is that if individuals use adaptive coping and are able to regain a sense of control over cancer challenges, they are then less likely to experience distress. In this sense, coping is not only a valuable explanatory concept regarding variability in response to stress, it can also serve as a portal for intervention, i.e., when adaptive coping skills are known, they can then be learnt. Although coping strategies may address and resolve the stressor, in some instances, such as a life-threatening illness like cancer, a favourable resolution might not be possible. At this point, the revised Lazarus & Folkman framework [46] proposes that coping then focuses on fostering positive emotions despite the presence of negative feelings engendered by the unresolved stressor. Considerable empirical evidence supports the variables postulated by this framework among individuals diagnosed with cancer and their partners [49-51]. Therefore, this framework provides a strong conceptual basis for *Coping-Together*. *Coping-Together* is expected to provide couples with the resources needed to manage physical and psychosocial cancer challenges that can be addressed directly, whereas for those stressors that cannot be immediately resolved, *Coping-Together* sustains meaning-based coping and fosters positive emotions. How the content of the *Coping-Together* intervention is expected to enhance the coping process is further depicted in Figure 1.

Despite the strengths of the Lazarus and Folkman framework, it mainly centers on the individual and the fundamental role that partners play is not explicit. Bodenmann’s framework of dyadic coping [47] extends Lazarus and Folkman’s framework by acknowledging the reciprocal nature of stress and coping within couples. This framework has been most frequently applied within the marital counselling literature, though it is becoming more popular within the cancer literature [52,53]. Broadly speaking, Bodenmann suggests two dyadic coping styles: positive dyadic coping (e.g., supportive communication; joint problem-solving) and negative dyadic coping (e.g., minimising partner concerns; sarcasm, mocking of concerns). Badr and colleagues [52] found that among couples coping with metastatic breast cancer, the use of positive dyadic coping was associated with greater adjustment than the use of negative dyadic coping.

Bandura’s self-efficacy theory posits that people are likely to engage in activities to the extent that they perceive themselves to be competent at those activities [48]. According to Bandura, there are four major sources of self-efficacy: 1) performing a task successfully (mastery experiences), 2) witnessing other people, especially those similar to oneself, successfully complete a task (vicarious experiences), 3) people could be persuaded to believe that they have the skills and capabilities to succeed (e.g., getting verbal encouragement from others to overcome self-doubt), and 4) addressing psychological responses that impact how a person feels about their abilities in a particular situation [54]. To address these sources of self-efficacy, the *Coping-Together* booklets provide step-by-step, practical guides to implement the coping strategies proposed; including behaviour therapy-based worksheets to encourage self-reflection, active learning, and promote application of coping skills to manage challenges confronted and quotes from other patients and partners reporting their success with the proposed coping strategies.

## Content

*Coping-Together* takes on a holistic approach to coping with cancer by including strategies to manage physical, social, psychological, and existential cancer-related challenges. The pilot version of *Coping-Together* includes a series of four booklets developed and qualitatively evaluated by the research team between 2009–2012 [55]. Each booklet addresses one of the following challenges and presents a range of coping strategies shown to be efficacious in managing these: 1) symptom management, 2) couples communicating effectively with health care professionals, 3) supporting each other, and 4) managing worries and emotions. Most available cancer information resources provide information on ‘What is cancer’ and what couples should or need to do, but the *Coping-Together* booklets provide information on ‘*How*’ couples can go about managing cancer challenges [34]. Multiple coping strategies are presented to couples to facilitate a personalised approach, where couples can ‘pick and choose’ and apply those that most closely correspond to their needs. Also, the booklets contain information about additional health care resources. For this pilot, the *Coping-Together* booklets are complemented by electronic media, including a relaxation CD and a 'Communicating effectively with health care professionals’ DVD. Participants will be able to use any or all of these resources at their own discretion and pace prior to collection of the outcome data, which will be 2 months following baseline measurements.

## Study aims and hypotheses

The overall study aims are to 1) examine the acceptability of the methods (e.g., recruitment, survey) to couples affected by prostate cancer and test the feasibility of providing *Coping-Together* and 2) collect preliminary data to investigate the short-term efficacy of *Coping-Together* on couples’ well-being. The study has two arms: 1) *Coping-Together* intervention arm and 2) Minimal ethical care (MEC) arm.

As this is a pilot study, it is not expected that there will be any significant statistical difference between the *Coping-Together* and MEC groups on primary and secondary outcomes; however, it is hypothesised that trends will be noted where *Coping-Together* couples will experience less anxiety (primary hypothesis), cancer specific distress and depression and more positive illness or care giving appraisal, self-efficacy, quality of life (QOL), relationship satisfaction and positive individual and dyadic coping (secondary hypotheses) at 2 months post-baseline compared to MEC couples.

## Methods/design

### Design

The proposed study is a multicentre, stratified, double-blind, two-group, parallel, randomized controlled trial to compare *Coping-Together* to MEC. The design of this study was guided by the Medical Research Council framework for developing and evaluating complex interventions [56] and the CONSORT statement [57].

### Sample

Based on other psycho-oncology feasibility studies [43,58,59] and suggestions by Hertzog [60], 35 couples/group will be recruited for this pilot.

#### Inclusion and exclusion criteria

Men diagnosed in the past 4 months with a primary, early-stage, prostate cancer, receiving or planning to receive treatment (including active surveillance), and their partner (spouse, boy/girlfriend, or de facto) will be invited to participate in the study. Additional inclusion criteria will be either the patient or their partner is identified by the Distress Thermometer (DT) as distressed (i.e., patient score of four or more) at the time of recruitment [36] and both need to be sufficiently fluent in English and cognitively able to complete surveys. Patient and partner consents are required for the couple to participate in this trial. As *Coping-Together* was designed to bring about change in how couples’ cope with cancer challenges and decrease anxiety, couples will be targeted soon after diagnosis, a time typically marked by many concerns and needs [61,62]. Moreover, as it is now well-recognised that ignoring patients’ or partners’ baseline distress can undermine the efficacy of an intervention (given the potential for floor effect) [63], distressed patients or partners are included in this study.

### Procedures

It is anticipated that most eligible men will be recruited through urologists’ private practices in Australia (New South Wales and South Australia). Weekly, urologists will identify patients meeting the medical (recent, primary, early-stage prostate cancer diagnosis and receiving/planning to receive treatment) and English fluency inclusion criteria. At their next appointment, interested patients will be invited to meet with the on-site research assistant to further discuss study participation and obtain a score on the DT. The research assistant will give interested and eligible participants a study package, which includes an information statement, a consent form, baseline survey, and a study pack to pass on to their partner. The team will follow-up one to two weeks later with non-responders. If the on-site research assistant is not present in the clinic, the urologists will give interested patients a study pamphlet and obtained verbal consent for a member of the team to contact them within the following week. The study will also be advertised through a range of media outlets, including radio, print, and online channels. It is anticipated that 370 patients will be approached to recruit the target sample size, assuming that 35% of patients or partners score four or more on the DT [64]; of those, 25% of couples will be ineligible, 30% will refuse participation, and 7% will be lost to follow-up [65]. This study has been approved by the University of Newcastle and the University New South Wales Human Research Ethics Committees.

### Randomization of group assignment

A computer-generated, randomization schedule with block lengths of variable size (4 and 6 couples) and stratified by recruitment source will be accessible to the main research assistant to allow assignment of a unique study ID to each couple as their consent forms/baseline surveys are received. Block randomisation is used to achieve balance in the allocation of couples across the MEC and *Coping-Together* groups and varying the block size reduces the chance of treatment allocation being known [66].

### C*oping-Together* and minimal ethical care (MEC) conditions

**Figure 2:**
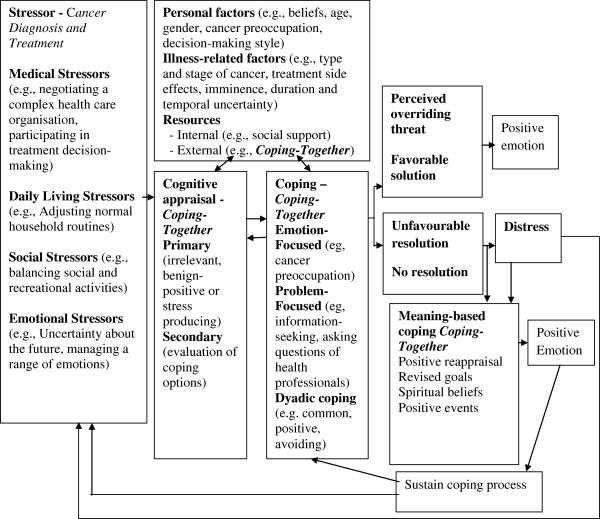
Study Timeline.

At recruitment, participants will be informed that they will be mailed one of two resources, without knowing which one is the study intervention. *Coping-Together* couples will receive the intervention material previously described within 2 weeks of returning a baseline survey (see Figure 2 – study timeline). One to two weeks thereafter, a member of the research team will phone them to orient them to *Coping-Together*. The intent of this brief orientation call (anticipated duration = 20 minutes) will be to ensure participants received the material, provide an overview of the content, and explore intended use of the resource. Couples will then receive, fortnightly, a 'Top Tips' sheet highlighting timely content of the booklets and a follow-up, telephone call (anticipated duration = 20 minutes) from a member of the research team to monitor the use of *Coping-Together* and other resources and answer questions pertaining to the materials received. Although the calls will be conducted by experienced, trained research assistants, the research assistants will not provide counseling, as they are not registered therapists. A script will be developed to guide the discussion.

As MEC couples also have elevated distress, and to blind participants to group allocation, this study will not employ a ‘no treatment’ control group. MEC couples will be mailed booklets from the ‘Understanding Cancer Series’ available at the Cancer Council New South Wales and a Cancer Council Helpline brochure, and will also receive initial and follow-up phone calls, comparable in intent and content to the one described for *Coping-Together* couples. All phone calls will be audio-recorded and coded to monitor topics discussed across intervention participants and ensure that counselling was not provided.

### Blinding

Participants will be told they will receive one of two information packs, but as they do not know which one is the study intervention and the study survey and contact with the research team is comparable across groups, they are blinded to whether they are in the MEC or *Coping-Together* group. The participants will also be blinded to study hypotheses. As it is expected that couples will mainly use *Coping-Together* at home, the chance of MEC participants being exposed to the intervention is minimal.

All contact with the couples will occur through the same research assistant(s) who will not be blinded to group allocation and will assign a study ID, randomise participants, send study packs, and conduct follow-up phone calls. A separate research assistant, who will have no contact with participants and will be blinded to group allocation, will enter the data. The chief investigators and the statistician will not be able to link study IDs (and surveys) back to a recruitment site or group and are therefore blinded to group allocation.

### Outcomes

As the development of *Coping-Together was* guided by the Lazarus & Folkman’s Stress and Coping framework (see Figure 1) [67], outcomes selected either characterise the coping process (appraisal, dyadic and individual coping strategies, and self-efficacy) or indicate the extent to which the coping process was successful in addressing stressors (anxiety, cancer distress, depression, quality of life, and relationship satisfaction).

#### Measures

Table 1 lists the primary and secondary outcomes and corresponding measures.

**Table 1:** ***Coping-Together *study outcomes and measures **

**Outcomes**	**Measures and Psychometrics**
**Primary Outcome**	
**Anxiety**	7-item HADS-Anxiety Subscale (patients and partners) [69] (α = .68-.93) [70]
**Secondary Outcomes**	
**Depression**	7-item HADS-Depression Subscale (patients and partners) [69] (α = .67-.90) [70]
**Cancer distress**	15-item Revised Impact of Event Scale (patients and partners; α = .78-.96) [71]
**Quality of Life (QOL)**	35-item Assessment of Quality of Life – 8 Dimensions Scale (AQoL-8D; patients and partners) [72]
	35-item Caregiver’s QOL Index-Cancer (partners) [73]
**Relationship satisfaction**	7-item Spanier Dyadic Adjustment Scale (patients and partners) [74] α = .89-.95) [75]
**Appraisal**	28-item Kessler Cognitive Appraisal of Health Scale (patients and partners [adapted]; α > .70) [76]
	33-item Mishel’s Uncertainty Scale (patients and partners; α = .64-0.91) [77]
	27-item Appraisal of Caregiving Scale (partners; α > .85) [78,79]
S**elf-efficacy**	17-item Cancer Self-Efficacy Scale (patients and partners; α = .97) [78]
	12-item Communication and Attitudinal Self-Efficacy Scale for Cancer (CASE-Cancer; patients and partners [adapted]; α = .76-.77) [80]
**Dyadic coping**	37-item Dyadic Coping Inventory (patients and partners; α = .63-.84) [81]
**Individual Coping**	28-item Brief COPE measures 14 individual-level coping strategies (patients and partners; α = .60-.90) [82]
Moderators*	
**Information obtained and information-seeking preferences**	25-item EORTC Quality of Life (QOL) – information module (patients and partners; α > 0.70) [83]
	45-item Profile of Preferences for Cancer Information (PPCI) (patients and partners [adapted]) [84]
**Readiness for self-directed learning**	39-item adapted version of the Learning Preference Scale (patients and partners [adapted]) [85]
**Problems experienced**	48-item adapted version of SupportScreen scale (patients and partners [adapted]) [86]

*Note.* Brackets indicate if patients and/or partners will complete the measure. * Questionnaires measuring potential moderators were included in the survey to reflect those that would be included in a survey for a larger trial to comprehensively examine the feasibility of the methods. However, the pilot is underpowered and moderators will not be analysed.

*Initial distress screening*: At the time of recruitment, the single-item DT will be used to screen patients’ level of distress. The DT asks patients to circle the number that best describes their overall distress using a visual analogue scale ranging from 0 = ‘no distress’ to 10 = ‘extreme distress’ [36]. The DT has convergent validity with the Hospital Anxiety and Depression Scale (HADS) [36], with a cut-off point of four typically resulting in optimal sensitivity and specificity [68].

*Primary outcome: Anxiety* is the primary outcome, as *Coping-Together* focuses on coping strategies to manage high levels of anxiety directly and/or cope with cancer demands known to trigger anxiety. *Anxiety* will be measured using the 7-item anxiety subscale of the Hospital Anxiety and Depression Scale (HADS-A) [69]. HADS-A is a reliable and valid measure of the severity of anxiety for patients with cancer and their partners and is often considered the ‘benchmark’ for validation of other anxiety measures [87-90].

#### S*econdary outcomes*

*Depression* will be measured using the 7-item depression subscale of the Hospital Anxiety and Depression Scale (HADS-D) [69].

Cancer specific *distress* will be measured with the Revised Impact of Event Scale [71].

*Quality of Life* (QOL) will be measured with the Assessment of Quality of Life – 8 dimensions (AQoL-8D). The AQoL8D is a newly developed health-related QOL instrument specifically for use with people with mental health problems and distress [72]. Also, the Caregiver’s QOL Index-Cancer will be used to assess caregiver’s mental and emotional burden, life disruption, positive adaptation and financial concerns [73].

*Relationship satisfaction* for partners and caregivers will be measured using the Spanier Dyadic Adjustment Scale (dyadic consensus, satisfaction, cohesion, and affective expression) [74], which is a widely used measure of satisfaction with intimate relationships.

*Primary illness appraisal* will be measured by the Kessler’s Cognitive Appraisal of Health Scale [76], Mishel’s Uncertainty in Illness Scale [77], and the Caregiving Illness Appraisal Scale [78,79].

*Secondary illness appraisal (self-efficacy)* will be measured by the Cancer Self-Efficacy Scale [78] and the Communication and Attitudinal Self-Efficacy Scale for Cancer (CASE-Cancer) [80].

*Individual and dyadic coping* will be measured by the Brief COPE [82] and the Dyadic Coping Inventory [81].

#### Moderators

Although this pilot is underpowered to examine the differential impact of the intervention across participant sub-groups, the pilot survey will include all measures that would potentially be considered for a larger trial to comprehensively examine the feasibility of the methods.

*Information obtained and information-seeking preferences* will be measured by the EORTC Quality of Life (QOL) – information module [83] and a tool developed by the first author – The Profile of Preferences for Cancer Information (PPCI) [84,91], respectively.

A cancer care diary developed by members of the research team will also be completed for the duration of the study to document *health professionals seen and health care resources utilised*.

*Readiness for self-directed learning* will be measured by items adapted from Guglielmino’s Learning Preference Scale [85].

*Problems experienced* will be measured by items adapted from the SupportScreen scale [86].

*Socio-demographic, disease and medical variables*, including date of diagnosis, treatment, symptom distress [92], age, and education, will also be measured.

### Data analysis

Intention-to-treat and per protocol analysis will be conducted separately for patients and partners. The primary outcome, anxiety (HADS-A) two months post baseline, will be analysed using ANalysis of COVAriance (ANCOVA). The main predictor variable in the ANCOVA model will be treatment group, and the participants’ baseline score will be included as a covariate. ANCOVA will also be used to explore the secondary outcome measures. Questionnaires measuring potential moderators were included in the survey to reflect those that would be included in a survey for a larger trial, and this contributes to a comprehensive examination of the feasibility of the methods; however, the pilot is underpowered and moderators will not be analysed.

### Process evaluation

Process evaluation will explore the intervention’s implementation and receipt [93]. Following their last data collection point, a process evaluation, semi-structured phone interview will be scheduled with consenting *Coping-Together* and MEC couples (consent obtained at baseline) to explore their views on, and opinions about, the *Coping-Together* or MEC material used and obtain feedback on the study’s process and procedures. Interviews will be audio-recorded and transcribed verbatim. For consistency, a couple’s process evaluation interview will be completed by the same research assistant that conducted their follow-up phone calls. The interview is anticipated to last 45–60 minutes. A script will be used to open and focus the discussion and prompts will be specified to help the interviewers to further elaborate on the study’s topics, if needed. All transcripts will be reviewed line-by-line and words, statements, or paragraphs pertaining to participants’ opinions about the *Coping-Together* or MEC material or the study will be coded. Interpretation and clustering of the codes will result in detecting themes and patterns. Transcripts will be coded by two research team members and emerging findings will be discussed at regular team meetings.

## Discussion

Partners’ anxiety rates exceed not only the Australian norm, but also those of cancer survivors themselves [5]. Hence, it is imperative that coping skills interventions target the couple as a unit and enhance both patients’ and partners’ ability to overcome cancer challenges. Recent reviews support the efficacy of coping skills training interventions in optimising patients' illness adjustment [26,94]. However, these interventions tend to be led by highly trained health professionals; considerably limiting their long-term sustainability, due to high cost and limited availability of qualified professionals, especially in non-metropolitan areas. In addition, it is recognised that patients who might benefit from conventional face-to-face psychosocial interventions do not access these, either by preference or because of geographical or mobility barriers [34,35]. Self-directed (also referred to as self-help or self-administered) interventions overcome these limitations, are acceptable to patients and efficacious in decreasing patients’ distress [34]. *Coping-Together* is a novel psychosocial intervention, as it targets both patients and their partners, translates up-to-date research on effective coping and renders it readily available to couples for their use where and when they need to, and actively engages couples in learning new coping skills. To our knowledge, *Coping-Together* is the first, self-directed intervention for couples affected by cancer.

### Implications

*Coping-Together* has the potential to have a direct impact on the psychological well-being of patients and their partners and to redress issues of access and equity for couples from regional, rural and remote areas, without burdening an already stretched oncology workforce. This pilot trial will investigate the feasibility and potential efficacy of *Coping-Together* in reducing the negative psychosocial impact of cancer on patients and their partners, which in turn will be used to design a larger trial that will not only examine the efficacy of *Coping-Together*, but also its direct cost and cost-effectiveness. Findings from this study will also contribute to the larger literature in advocating for psychosocial care in the acute post-diagnostic phase and in identifying individual and couple-level factors that contribute to patients’ and partners’ coping, anxiety, and use of self-directed resources. If future studies support the efficacy of *Coping-Together*, the intervention’s low production cost and self-directed nature will contribute to its ready integration into existing supportive care infrastructures for patients diagnosed with prostate cancer and their partners.

## Abbreviations

NHMRC: National Health and Medical Research Council; PST: Problem-solving therapy; MEC: Minimal ethical care; QOL: Quality of life; DT: Distress Thermometer; HADS: Hospital Anxiety and Depression Scale; AQoL-8D: Assessment of Quality of Life – 8 dimensions; CASE-Cancer: Communication and Attitudinal Self-Efficacy Scale for cancer scale; PPCI: Profile of Preferences for Cancer Information; ANCOVA: ANalysis of COVAriance.

## Competing interests

The authors declare that they have no competing interests.

## Authors’ contributions

SL conceived the study, participated in its design, led the development of the *Coping-Together* resource, and drafted the manuscript. AG, JT, PV and KK participated in the design of this study, provided critical feedback on *Coping-Together*, and provided feedback on the draft of this manuscript. PM participated in the design of this study and provided feedback on the draft of this manuscript. All authors have read and approved the final manuscript.
